# A Voltage Dependent Non-Inactivating Na^+^ Channel Activated during Apoptosis in *Xenopus* Oocytes

**DOI:** 10.1371/journal.pone.0088381

**Published:** 2014-02-28

**Authors:** Ulrika H. Englund, Jens Gertow, Katarina Kågedal, Fredrik Elinder

**Affiliations:** Department of Clinical and Experimental Medicine, Linköping University, Linköping, Sweden; Universidad de Castilla-La Mancha, Spain

## Abstract

Ion channels in the plasma membrane are important for the apoptotic process. Different types of voltage-gated ion channels are up-regulated early in the apoptotic process and block of these channels prevents or delays apoptosis. In the present investigation we examined whether ion channels are up-regulated in oocytes from the frog *Xenopus laevis* during apoptosis. The two-electrode voltage-clamp technique was used to record endogenous ion currents in the oocytes. During staurosporine-induced apoptosis a voltage-dependent Na^+^ current increased three-fold. This current was activated at voltages more positive than 0 mV (midpoint of the open-probability curve was +55 mV) and showed almost no sign of inactivation during a 1-s pulse. The current was resistant to the Na^+^-channel blockers tetrodotoxin (1 µM) and amiloride (10 µM), while the Ca^2+^-channel blocker verapamil (50 µM) in the bath solution completely blocked the current. The intracellular Na^+^ concentration increased in staurosporine-treated oocytes, but could be prevented by replacing extracellular Na^+^ whith either K^+^ or Choline^+^. Prevention of this influx of Na^+^ also prevented the STS-induced up-regulation of the caspase-3 activity, suggesting that the intracellular Na^+^ increase is required to induce apoptosis. Taken together, we have found that a voltage dependent Na^+^ channel is up-regulated during apoptosis and that influx of Na^+^ is a crucial step in the apoptotic process in *Xenopus* oocytes.

## Introduction

Programmed cell death, apoptosis, is an essential process in the development and the sculpturing of the growing organism. Apoptosis is a cascade process involving a sequence of biochemical reactions. Altered functions in the apoptotic cascade, for example caused by mutations, can lead to diseases. Much research carried out in the field of apoptosis has focused on biochemical alteration in intracellular signalling cascades, where pro-apoptotic proteins and proteases are activated, mitochondrial proteins released, and DNA degradation occurs [1]. However, the initial steps triggering the apoptotic process are less known. Alterations in the intracellular ion concentrations have been one such suggested initial event [2], [3]. A number of ion channels open during the early steps of the apoptotic process and block of the channels also prevents or delays the apoptotic process. Apoptotic stimuli, such as staurosporine (STS) or serum deprivation has been shown to activate K^+^ channels [4]. Opening of K^+^ channels leads to an efflux of K^+^ ions and a decrease in the intracellular K^+^ concentration [4]–[7]. The decreased K^+^ concentration has been shown to facilitate activation of caspases and apoptosis-associated nucleases. Consequently, block of K^+^ channels prevents apoptosis [4], [8], [9]. Opening of Na^+^ channels, leading to an increased Na^+^ influx and intracellular Na^+^ concentration also triggers apoptosis. Consequently block of Na^+^ channels prevents apoptosis [2], [3], [10]–[14]. Finally, also Cl^−^ channels have been linked to apoptosis. STS opens of a large-conductance Cl^−^-conducting anion channel [10], [15], [16], which has been suggested to be identical to the mitochondrial voltage-dependent anion channel, VDAC. Because the Cl^−^ equilibrium potential and consequently the intracellular Cl^−^ concentration normally follows the resting potential [17], opening of the channel is not expected to alter the Cl^−^ concentrations. However, opening of Cl^−^ channels is possibly an essential step in combination with opening of K^+^ channels [4], [15], [18]. Block of these Cl^−^ channels has also been shown to prevent apoptosis.

Thus, alterations in intracellular ion concentrations are early steps in the apoptotic process. Despite this crucial role, still very little is known about the details of the role of ion channels and intracellular concentrations in apoptosis. Most cells are small and less suitable for biophysical investigations. Therefore, in the present investigation, we utilized large oocytes from the clawed toad *Xenopus laevis* as a model cell. These oocytes are easy to investigate with electrophysiological techniques, they have been shown to undergo classical apoptosis [19], [20], and was recently shown that intracellular ion concentrations can be measured by nanoprobes [21]. STS activates caspase-3 and decreases the intracellular K^+^ concentration, but this K^+^ concentration alteration was shown not to be obligatory for the apoptotic process [22]. The specific purpose of the present investigation was to explore if the *Xenopus* oocyte alters its electrophysiological characteristics during STS-induced apoptosis, and if so, if the apoptotic process could be affected by preventing ion fluxes.

## Results

### Staurosporine induced apoptosis in *Xenopus* oocytes

**Figure 1:**
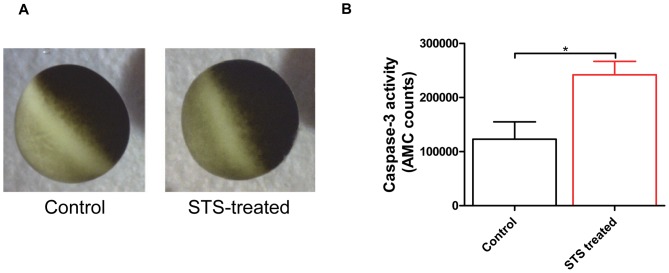
Apoptosis in *Xenopus laevis* oocytes. **A**. Morphological changes of *Xenopus laevis* oocytes before (Control) and after 6 hours of treatment with 20 µM staurosporine (STS-treated). **B**. Measurement of caspase-3 activity in control oocytes (black, 120,000±30,000, *n = *9) and staurosporine (1 µM) treated oocytes (red, 240,000±25,000, *n = *23). Statistical analysis is unpaired t-test (* P<0.05).

*Xenopus* oocytes have previously been shown to increase its caspase-3 activity when challenged by 1 µM STS [22]. However, little is known of the details about electrophysiological properties and intracellular ion concentrations. Here, we wondered if STS also alters morphological characteristics and other signs of apoptosis. Figure 1A shows microscope pictures of oocytes incubated in a control solution and in 20 µM STS for 6 hours. The STS-exposed oocyte showed typical signs of apoptosis: A more diffuse line between the animal and vegetable pools, as compared to control oocytes. 1 µM STS also caused these changes but then a longer exposure time was needed. We also measured caspase-3 activity in the oocytes. In contrast to many other cells, the control oocytes showed a high and variable level of caspase-3 activity. Despite this high level in control oocytes, 1 µM STS for 6 hours almost doubled the caspase-3 activity (Fig. 1 B, control: 120,000±30,000 *n* = 9, black; STS: 240,000±25,000 *n* = 23, red. Unpaired t-test p<0.05). Thus, the *Xenopus* oocyte seems to show normal signs of apoptosis.

### Activation of a voltage-gated ion channel during apoptosis

**Figure 2:**
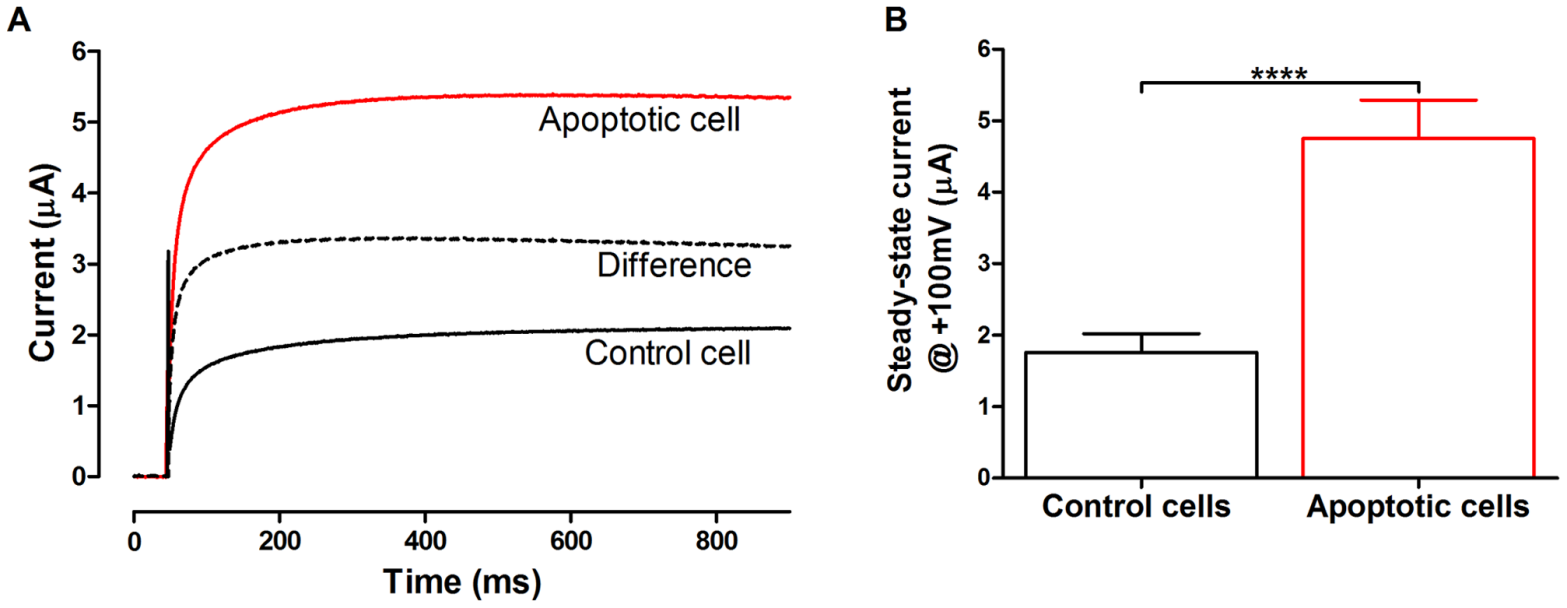
Voltage-gated ion channel activated during apoptosis. **A**: Electrophysiological properties of an outward current in control oocytes (black) and staurosporine (1 µM) treated oocytes (red) in *Xenopus* oocytes at +100 mV. The difference between the current in control and STS-treated oocytes is also plotted (dashed line). **B**. Mean steady-state current at +100 mV in control (1.8 µA±0.5 µA, *n* = 17) and apoptotic oocytes treated with 1 µM STS (4.8 µA±0.3 µA, *n* = 13). Steady state currents at +100 mV are significantly larger in the apoptotic oocytes than in controls (**** P<0.0001). Statistical analyses are mean ± SEM and unpaired t-test. Recordings were done in 100Na solution.

To explore possible alterations in ion channel activity of the oocytes we used the two-electrode voltage-clamp technique to measure transmembrane ion currents. Control oocytes have several types of ion channels but normally the activity is low, making them suitable as an expression system for exogenous channels [23]. Figure 2A shows typical current recordings at +100 mV from control and STS-induced apoptotic oocytes, respectively. The current reached steady state after 300–400 ms and did not show any sign of inactivation during a 1-s pulse. To isolate the STS-induced current component we also plot the difference (dashed trace), by subtracting the current in control cells (black trace) from the current in apoptotic cells (red trace). The difference current is similar to control and apoptotic current but differs in one aspect: it reaches a maximum at about 300–400 ms and declines about 5% during the 1-s pulse. This suggests, either that (i) the current in control oocytes is different from the current that is induced during apoptosis or that (ii) the current in control oocytes is a mix of two or more currents: one that is slowly activating during the entire 1-s pulse and one that is slowly inactivating and which is enhanced during apoptosis. As will be shown later on in this work, there is a slowly activating ion current different from the STS-induced current, thus supporting the second hypothesis. The steady-state current at +100 mV (measured 300 ms after onset of the pulse) was 1.8±0.3 µA (*n* = 17) in control oocytes. After 6 hours exposure to 1 µM STS the outward current was increased almost 3-fold to 4.8±0.5 µA (*n* = 13) (Fig. 2B).

### Properties of the up-regulated ion current

**Figure 3:**
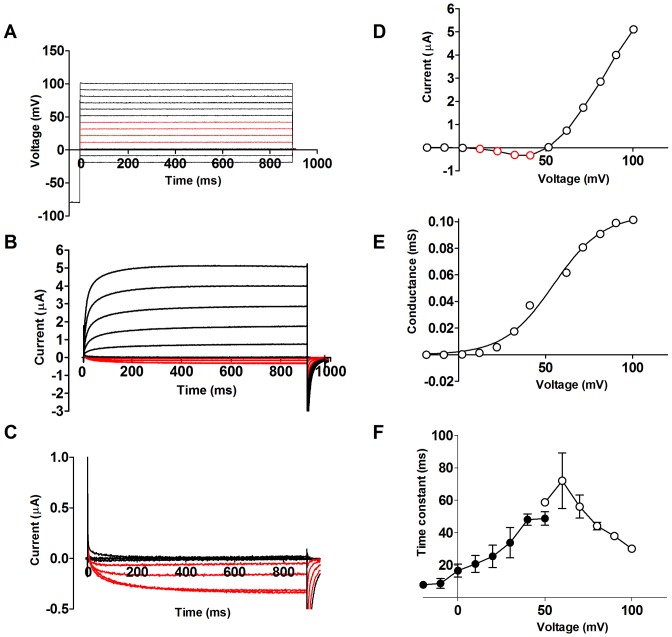
Properties of the STS-induced current. **A**. The voltage pulse protocol. **B**. Typical corresponding currents, showing inward current at intermediate voltages (see red traces). The recording has been corrected for leak conductance of 1.4 µS. **C**. Magnification from **B**. The inward currents between +10 and +40 mV are shown in red. **D**. Steady-state current vs. voltage from **B**. **E**. Typical *G*(*V*) curve for the STS-induced currents. **F**. Time constants for the fast activation component from a double exponential fit (open symbols, *n = *5), and the single exponential closing of the studied current (closed symbols, *n = *8).

To explore the identity of the channel activated during apoptosis, we first investigated the kinetic properties of the channel. Figure 3A and B show a typical family of currents associated with voltage steps between −20 and +100 mV from a holding voltage of −80 mV. At −20 to +10 mV no current was activated. At +10 to +40 mV the current was inward (Fig 3C, red traces). Voltage steps to +60 mV and above generated large positive (outward directed) currents. After 900 ms the membrane voltage was changed to 0 mV. This step produced a large inward going current and closed the channel (Fig. 3B and C). The steady-state current plotted versus membrane voltage produced a curve crossing the x-axis at +55±4 mV (*n* = 7) (Fig. 3D). This positive reversal potential suggests that the channel conducts Na^+^ rather than K^+^ or Cl^−^ ions. The large outward going current also makes it unlikely that it is a Ca^2+^ channel because the intracellular Ca^2+^ concentration is very low [24]. Figure 3E shows the steady-state conductance-versus-voltage, *G*(*V*), calculated from Eq. 1 for STS-induced currents. The midpoint voltage was +55±2 mV (*n* = 3), and the apparent gating charge *z*_g_ was 2.0±0.2 (*n* = 3) (Eq. 2). This suggests that the voltage sensor of the channel moves at least 2 charges before opening the channel. The closing kinetics was roughly exponential and the time constant for STS-treated oocytes is shown in Figure 3F (closed symbols, *n* = 8). Opening kinetics is more complicated with a fast and a slow component. The slow component probably represents another current (see previous section). Figure 3F shows time constants for the opening kinetics of the fast component (open symbols, *n* = 5).

### The voltage-gated ion channel is a Na^+^ channel

**Figure 4:**
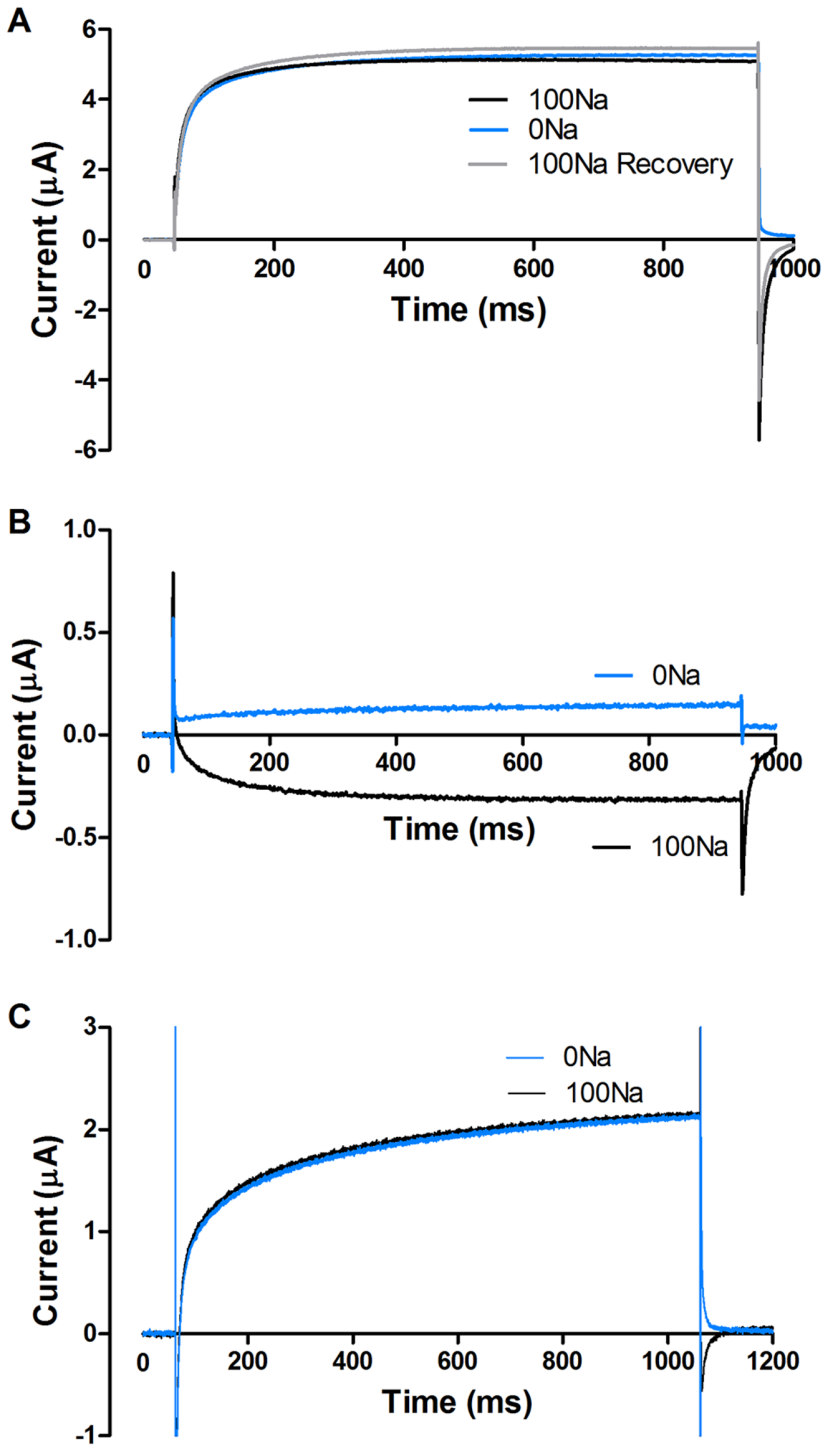
The voltage-gated ion channel is a Na^+^ channel. **A**. Tail currents measured at 0 mV after a prepulse to +100 mV demonstrating that the inward tail current in 100Na solution (black, *n = *4) is abolished in 0Na solution (blue) in STS-treated oocytes. Switching back to 100Na recovered the inward current (grey). **B**. The same recording but at +30 mV showing inward current in control solution (black) and outward in 0Na (blue).**C**. The inward tail currents measured in control oocytes are also abolished when 100Na (black) is replaced with 0Na (blue, *n = *3) in the extracellular solution.

The analysis above suggested that the channel was selective for Na^+^. To investigate this hypothesis further, we removed Na^+^ from the extracellular solution. Changing the extracellular solution from 100 mM Na^+^ (100Na) to 0 mM Na^+^ (0Na), where Na^+^ is completely replaced by K^+^, showed that although a similar outward going current was present in both solutions, the inward going tail currents (after 950 ms) in 100Na solution disappeared in 0Na (Fig. 4A). This alteration in tail current was completely reversible when Na^+^ was reintroduced (Fig. 4A, *n* = 4). A similar type of recording, where the channel is activated at +30 mV, shows an inward current in 100Na solution but an outward current in the 0Na solution (Fig. 4B). Also here the inward going tail is gone at 0Na. In control oocytes, the inward going tail current was smaller, but removal of extracellular Na^+^ abolished the inward tail (Fig. 4C). All differences between the recordings with and without Na^+^ must be mediated by Na^+^; the current could not be carried by either Cl^−^ or Ca^2+^, since their concentrations were not changed. It could not be carried by K^+^ because this would increase the tail current rather than decreasing it. The inward going tail current relative the outward going steady-state current in control oocytes is smaller compared to STS-treated oocytes. The reason for this is most likely that the contribution of another current, described later in this work, is relatively larger in control oocytes. Taken together, these experiments suggested that the apoptosis-induced ion channel is Na^+^ selective.

### Pharmacology of the Na^+^ channel

**Figure 5:**
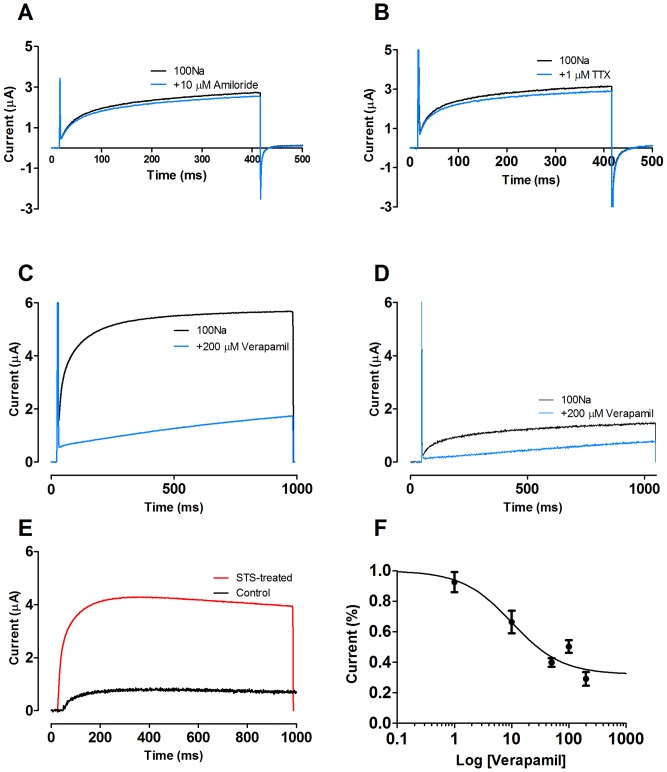
Pharmacology of the Na^+^ channel. **A**. 10 µM amiloride (blue) does not block the STS-induced current at a voltage-clamp step to +100 mV. **B**. 1 µM Tetrodotoxin (blue) does not block the STS-induced current at a voltage-clamp step to +100 mV. **C**. 200 µM verapamil (blue) blocks the fast activation current in STS-treated oocytes, leaving a slowly activating current. **D**. 200 µM verapamil (blue) blocks the fast activating current in control oocytes. **E**. Difference plots from **C** and **D** showing the verapamil sensitive Na^+^ current in STS-treated (red) and control (black) oocytes. **F**. Dose-response curve for the effects of verapamil (n = 3–5). IC_50_ = 10.1 µM with the Hill coefficient  = 1.

The above described Na^+^ channel is atypical because it does not show any prominent inactivation during a 1-s pulse to +100 mV (Fig. 3B). Most Na^+^ channels show a fast inactivation [25]. Among the 10 Na^+^ channels found in the human genome only one lacks fast inactivation [26]. Unfortunately, it was not possible to apply longer, 10-s, pulses to explore if there is a clear inactivation in the STS-induced currents because such long pulses activated other ion channels in control cells that irreversibly altered the electrophysiological properties of the oocytes. To further characterize the ion channel, we explored the effect of several classical Na^+^ channel blockers. The channel was insensitive to high concentrations of either amiloride (known to block weakly voltage dependent epithelial Na^+^ channels; Fig. 5A) or of tetrodotoxin (known to block most, but not all, of the voltage-gated Na^+^ channels; Fig. 5B). Instead we explored the promiscuous Ca^2+^-channel blocker verapamil which also blocks Na^+^ and K^+^ channels [27]–[30]. 200 µM verapamil almost completely abolished the current (Fig. 5C), and only left a slowly activating component which most likely is another type of channel. 200 µM verapamil had a similar effect on control oocytes (Fig. 5D), but the relative reduction was, however, much smaller. The most likely explanation is that both control and STS-treated oocytes have a slowly activating component of about the same size while incubation in STS up-regulates the Na^+^ current with much faster activation kinetics.

To explore this component in more detail we plotted the difference between currents (from Fig. 5C and D) with and without verapamil (Fig. 5E). These verapamil difference currents not only have almost identical time courses, they are also almost identical to the STS-induced current (Fig. 2A). This suggests that the 3-fold increase in total current (Fig. 2) in STS-treated oocytes is an underestimation of the increase in the slowly non-inactivating Na^+^ current, it should be closer to five-fold (Fig. 5E). Because the STS-induced current (Fig. 5E) peaked at about 300 ms after onset of the voltage pulse we measured the current reduction effect at this time point. 200 µM verapamil reduced the current by about 70%. Both 50 µM and 100 µM had similar effects (Fig. 5F) suggesting that saturation had been reached at 200 µM. The remaining 30% which also had a much slower opening kinetics is believed to be of other origin. 50% of full block effect was reached at 10 µM (Fig. 5F).

### Comparison of the non-inactivating Na^+^ current in STS-treated and control oocytes

**Figure 6:**
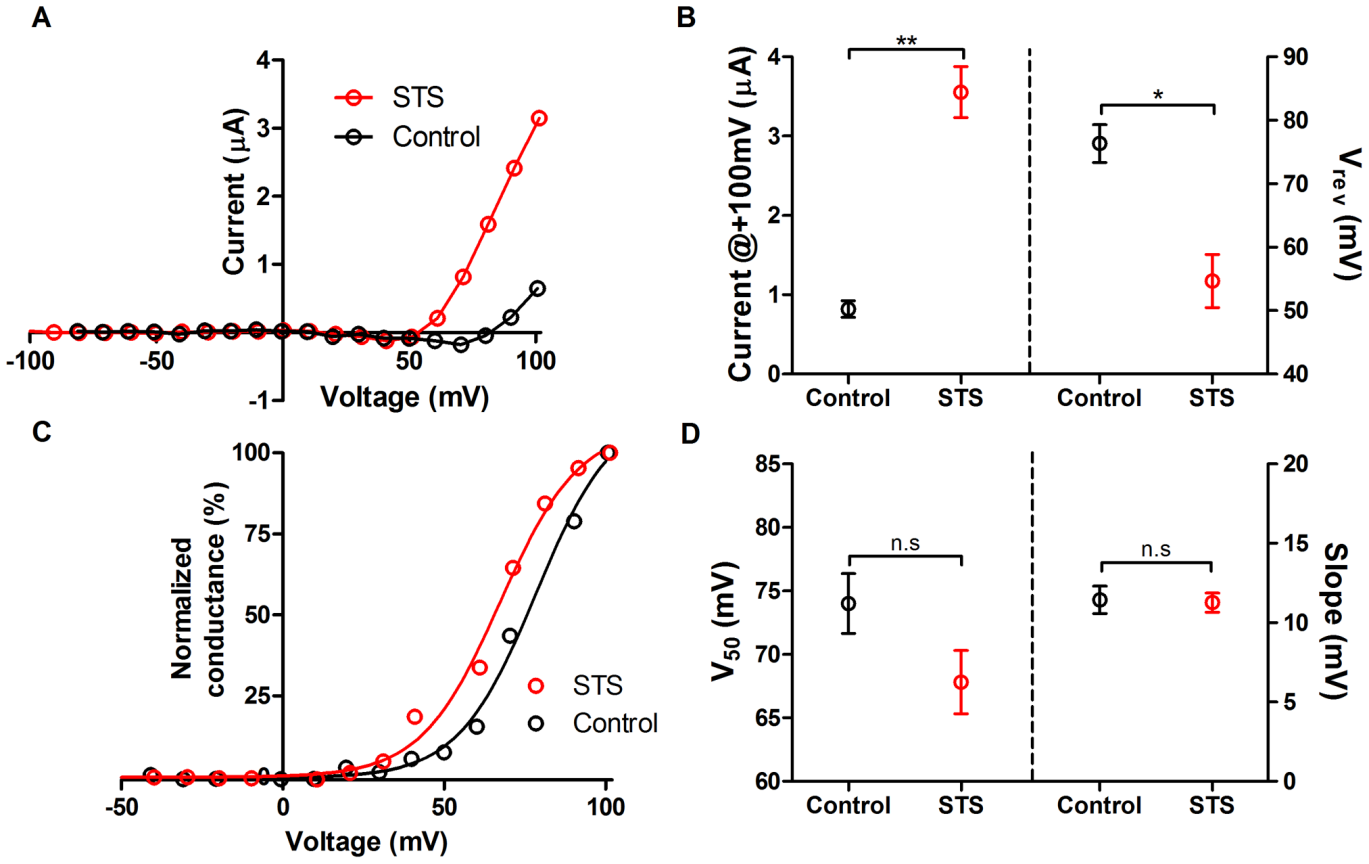
Comparison of Na^+^ currents in STS-treated and control oocytes. **A**. Typical Steady-state current versus voltage curve for the fast activated verapamil-sensitive Na^+^ current after subtraction of the slow activated verapamil-insensitive current in STS –treated oocytes (red) and control oocytes (black). **B** Mean steady-state current at +100 mV (left) after subtraction of the slow activated current for STS-treated oocytes (red, 3.55±0.3 µA, *n* = 3) and control oocytes (black, 0.8±0.1 µA, *n* = 3). Mean reversal potential (V_rev_, right) after subtraction of the slow activated current for STS-treated oocytes (red, +55±7 mV, *n* = 3) and control oocytes (black, +76±6 mV, *n* = 3). Statistical analyses are mean±SEM and unpaired t-test (* P<0.05 and ** P<0.01) **C**. Normalized *G*(*V*) curves for the fast activated Na^+^ current after subtraction of the slow activated current in STS-treated (red) and control oocytes (black). **D** Mean V_50_ (left) after subtraction of the slow activated current for STS-treated oocytes (red, +68±4 mV, *n* = 3) and control oocytes (black, +74±4 mV µA, *n* = 3). Mean slope (Slope, right) after subtraction of the slow activated current for STS-treated oocytes (red, 11.2±1.1 mV, *n* = 3) and control oocytes (black, 11.4±1.5 mV, *n* = 3). Statistical analyses are mean±SEM and unpaired t-test.

To compare the non-inactivating Na^+^ current in control oocytes with that in STS-treated oocytes we investigated the difference plots from recording with and without 200 µM verapamil. Figure 6A shows typical *G*(*V*) curves of the verapamil-blocked current from control and STS-induced apoptotic oocytes. The amplitude at +100 mV is increased from 0.8±0.1 µA (*n* = 3) to 3.6±0.3 µA (*n* = 3) during STS treatment (p<0.01), and the reversal potential is shifted from +76±6 mV (*n* = 3) to +55±7 mV (*n* = 3) during STS treatment (p<0.05) (Fig. 6B). The Na^+^ conductance at 100 mV increased 2.7-fold, from 0.03±0.004 mS (*n* = 3) to 0.08±0.01 mS (*n* = 3), during STS-treatment. However, there is no significant difference in V_50_ or slope of the *G*(*V*) curves (Fig. 6C-D).This suggests that the Na^+^ current is present in control cells and that it is up-regulated in the apoptotic oocytes with no alterations in its properties.

### The role of Na^+^ influx for the intracellular Na^+^ concentration, the Na^+^ conductance, and apoptosis

If a Na^+^ current is activated during apoptosis this might increase the intracellular Na^+^ concentration, which could be a critical event in triggering the apoptotic process. To explore this we tried to reduce the Na^+^ influx. The most obvious way to reduce the influx was to block the channel by verapamil. Unfortunately, the oocytes did not tolerate a six-hour incubation in 1 µM STS together with 200 µM verapamil; the oocytes became fragile and leaky, and impossible to handle. Instead, to prevent the Na^+^ influx, we removed Na^+^ from the extracellular solution. Extracellular Na^+^ chloride was substituted by either choline chloride or tetramethylammonium chloride (TMA) [31] during the six hour incubation period. The choline treatment, but not the TMA treatment, was tolerated. Thus, for choline chloride treated cells electrophysiological recordings could be carried out.

**Figure 7:**
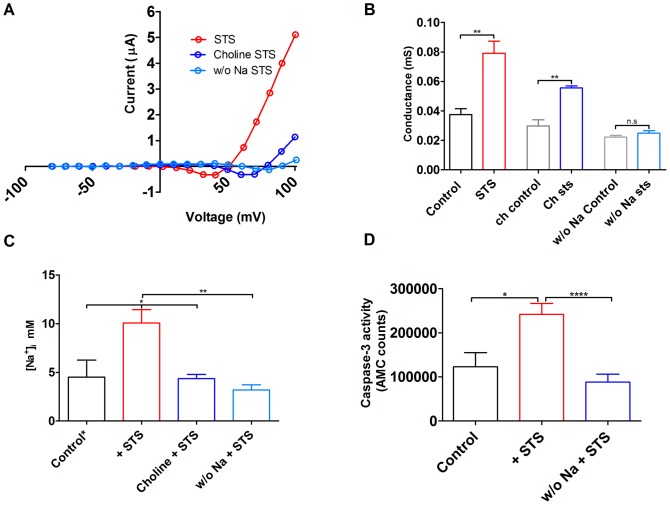
Role of Na_x_ and intracellular ions during apoptosis. **A**. Steady-state current vs. voltage for oocytes treated with 1 µM STS in MBS solution (red), 1 µM STS in MBS solution where NaCl had been replaced by ChCl (dark blue) and 1 µM STS in MBS solution where NaCl had been replaced by KCl (light blue, w/o Na). The electrophysiological measurements were done in 100Na solution. Replacing extracellular Na^+^ with either choline^+^ or K^+^ shifted the reversal potential from +50 mV to +80 mV, and +90 mV, respectively. **B**. The conductance at +100 mV increased in STS-treated (red, 0.08±0.01 mS, *n* = 5) compared to control oocytes (black, 0.04±0.004 mS, *n* = 5). Replacing extracellular Na^+^ with choline^+^ did not affect the Na^+^ conductance neither in control oocytes (grey, 0.03±0.004 mS, *n* = 6) nor in oocytes treated with 1 µM staurosporine (blue, 0.06±0.001 mS, *n* = 3). Replacement with KCl instead of NaCl did not increase the conductance for STS-treated oocytes (light blue, 0.02±0.001 mS, *n* = 4) compared to control (light grey, 0.02±0.002 mS, *n* = 4). Statistical analyses are mean±SEM and unpaired t-test (** P<0.01). **C**. Calculated intracellular Na^+^ concentrations, using Nernst's equation (Eq. 3), for oocytes treated with 1 µM STS in MBS solution (red, 10.1±1.4 mM, *n* = 4), 1 µM STS in MBS solution where NaCl had been replaced by ChCl (blue, 4.3±0.4 mM, *n* = 3) and 1 µM STS in MBS solution where NaCl had been replaced by KCl (light blue, 3.2±0.6 mM, *n* = 4). Intracellular Na^+^ concentration for control oocytes (control*, black, 4.5±1.8 mM, *n* = 3) were taken from other reports [32]–[34]. Statistical analyses are mean±SEM and one-way ANOVA test with Bonferroni *post hoc* tests (* P<0.05, ** P<0.01) **D**. Low extracellular Na^+^ (replaced by K^+^) prevented caspase-3 activation (control = 120,000±30,000, *n = *9, STS = 240,000±25,000, *n = *23 and w/o Na+STS = 90,000±18,000, *n = *19).Statistical analyses are mean±SEM and one-way ANOVA test with Bonferroni *post hoc* tests (* P<0.05, **** P<0.0001)

Most significantly, the reversal potential in STS-treated oocytes shifted from +55±7 mV (*n* = 3; red curve in Fig. 7A) to 78±4 mV (*n* = 3; dark blue curve in Fig. 7A) in choline^+^ incubation compared to normal extracellular solution, indicating less intracellular Na^+^ in the choline-incubated cells. The reversal potential in control oocytes (+76±6 mV, *n* = 3) is similar to choline/STS-treated cells (78±4 mV, *n* = 3). In contrast, the conductance was significantly increased, almost doubled, for STS-treated oocytes compared to control oocytes, independent of the identity of the extracellular cation (Na^+^ or choline^+^; Fig. 7B). This suggests that the increase in intracellular Na^+^ is not critical for the increased Na^+^ conductance. Substitution of Na^+^ by K^+^ during incubation also kept the reversal potential close to that for control oocytes (light blue curve in Fig. 7A, labelled w/o Na STS, 87±8 mV, *n* = 4). However, STS did not increase the conductance at +100 mV in the KCl experiments (w/o Na in Fig 7B), but these data are difficult to judge because of the closeness to the reversal potential. A summary of all conductance data are shown in Fig 7B (Control = 0.04±0.004 mS, *n* = 5, black; STS = 0.08±0.01 mS, *n* = 5, red; Choline control = 0.03±0.004 mS, *n* = 6, grey; Choline + STS = 0.06±0.001 mS, *n* = 3, blue; w/o Na control = 0.02±0.001 mS, *n* = 4, light grey; w/o Na + STS = 0.02±0.002 mS, *n* = 4, light blue).

Calculation by Nernst's equation (Eq. 3) shows that removal of extracellular Na^+^ during the STS-incubation period also decreased the intracellular Na^+^ concentration to control levels (Fig. 7C, Control* = 4.5±1.8 mM, *n* = 3, black; STS = 10±1.4 mM, *n* = 4, red; Choline + STS = 4.4±0.4 mM, *n* = 3, dark blue; w/o Na + STS = 3.2±0.6 mM, *n* = 4, light blue. P<0.05 (*) and 0.01 (**)). The intracellular Na^+^ concentration from the present investigation is close to data from other studies measured by different techniques in enzymatically defolliculated oocytes (5–6 mM) [32]–[34]. The caspase-3 activity increased in STS-treated oocytes (Fig. 2B), and this effect was effectively blocked when the oocytes were STS-treated in a solution without Na^+^, where Na^+^ had been replaced by K^+^ (Fig. 7D, Control = 120,000±30,000 *n* = 9, black; STS = 240,000±25,000 *n* = 23, red; w/o Na + STS = 90,000±18,000, *n* = 19, blue. P<0.05 (*) and 0.0001 (****)). To summarize these experiments, STS increases the Na^+^ conductance which leads to in increased Na^+^ influx. This Na^+^ influx leads to an increased caspase-3 activity.

## Discussion

**Figure 8:**
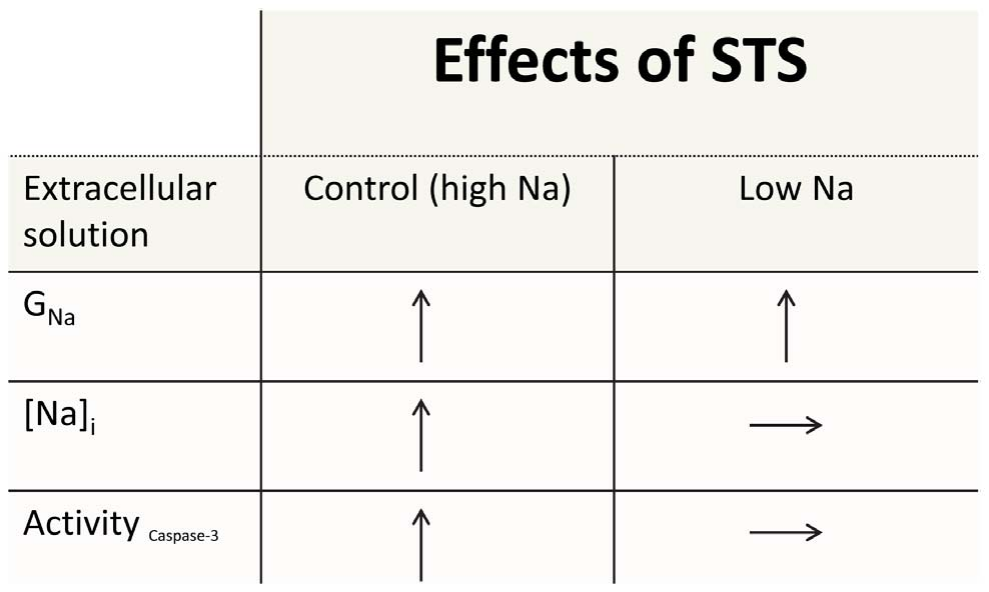
Summary of effects of staurosporine on *Xenopus* oocytes. See text for explanation.

In the present investigation we have found that an endogenous Na^+^ channel in *Xenopus* oocytes is up-regulated during STS-induced apoptosis and that the Na^+^ influx is essential for the apoptotic process. Figure 8 summarizes the main findings in this article. STS increased the Na^+^ conductance, increased the intracellular Na^+^ concentration, and increased the caspase-3 activity in normal physiological solutions. By lowering the extracellular Na^+^ concentration, STS could still increase the Na^+^ conductance, but failed to increase the intracellular Na^+^ concentration and thereby preventing caspase-3 activation and apoptosis.

### Identity of the Na^+^ channel

The described ion channel activated during apoptosis is an atypical Na^+^ channel. Most voltage-gated Na^+^ channels have a steep voltage dependence (slope corresponds to 4 elementary charges), open at voltages around −30 mV, activate fast (<1 ms), inactivate quickly (<10 ms), and are highly sensitive to tetrodotoxin [25]. The Na^+^ channel described here has a slightly shallower voltage dependence and activates slightly slower (about 10 ms). However, the other parameters are completely different: There is no clear inactivation during 1 s pulses. The channel is activated at much more positive voltages (around +50 mV), and it is not sensitive to tetrodotoxin. These properties do not fit to any conventional Na^+^ channel described in the human or rat genomes. Previous reports show at least four different endogenous Na^+^ currents in oocytes: 1. Na^+^ channels sensitive to amiloride [35], 2. Na^+^ currents induced by an intracellular acidification induced by NH_4_^+^ [36], 3. Na^+^ channels activated by ATP [37], and 4. Na_x_ channels activated by a positive voltages [32], [38]. Even though we have not tested for intracellular acidification and NH_4_^+^, we found that Na_x_ is a strong candidate for the channel described in the present investigation. Na_x_ has been reported to be blocked by the local anaesthetics lidocaine; 1 mM lidocaine reduces the Na_x_ current by about 75% [39]. Therefore, as a further test of the Na_x_ hypothesis, we also tested lidocaine on the STS-induced current. 1 mM lidocaine blocked the verapamil-sensitive fraction of the current by 67±5% (n = 3), thus supporting that the STS-induced current is identical to the Na_x_ current.

### Role of Na_x_ and intracellular ions during apoptosis

The Na_x_ channel conductance is up-regulated during apoptosis and this up-regulation could be caused either by altered expression or by altered channel properties. The genome for *Xenopus laevis* is not yet completely sequenced, so expression studies are not possible to do. However, because we have not been able to detect any alterations in channel properties in STS-treated oocytes compared to control oocytes, we think an increased expression is the most likely explanation for the increased conductance.

We have also shown that prevention of Na^+^ influx prevents apoptosis. Thus, it is possible that the Na^+^ influx through Na_x_ is an important event in the apoptotic process. One possibility is that Na^+^ ions directly affects the caspases. Another possibility is that the increased intracellular Na^+^ concentration leads to an increased intracellular Ca^2+^ which consequently affects the apoptotic process. Increased intracellular Ca^2+^ has been suggested to trigger apoptosis in different cell types [40], [41]. Normally intracellular Ca^2+^ is pumped out through a Na^+^/Ca^2+^ exchanger (NCX exchangers) [42]. An important consequence of increasing the intracellular Na^+^ concentration during apoptosis could be to change the intracellular concentration of other ions [2], [3]. The NCX exchanger (NCX1 and NCX3) is known to be expressed in oocytes and in physiological conditions intracellular Ca^2+^ is exchanged with Na^+^ in a 1∶3 ratio [42], [43]. However, an increase of intracellular Na^+^ could reverse the direction of Ca^2+^ to an inward current and thereby increasing the intracellular Ca^+2^ concentration. So the change of intracellular ion composition, especially Na^+^ effect on Ca^2+^ concentration, could be a pathway that leads to cell death [14], [40], [41], [44].

There are many reports where high concentration of extracellular K^+^ inhibits apoptosis [4]–[7]. In line with this, we found in a previous investigation that intracellular K^+^ decreases during STS-induced apoptosis in *Xenopus* oocytes, but we also found that maintaining the intracellular concentration of K^+^ did not prevent apoptosis [22]. Together with the data in the present investigation, we suggest that an increase in intracellular Na^+^ rather than a decrease in intracellular K^+^, is crucial for the apoptotic process in *Xenopus laevis* oocytes, and consequently, it is not the *high* extracellular K^+^ concentration but the *low* extracellular Na^+^ concentration that prevents apoptosis. This also fits with other reports on the role of intracellular Na^+^ [14], [44], [45].

## Materials and Methods

### Ethics statement

The animal experiments in this study were approved and performed in accordance with the guidelines from the European parliament and Community Council Directive (2010/63/EU) and the Swedish National Board for Laboratory Animals under the ethical permit Dnr: 125-10, which was approved by Linköpings djurförsöksetiska nämnd (the local animal research ethical committee in Linköping).

### Cell preparation

Oocytes were surgically collected from adult female frogs of the species *Xenopus laevis*. Oocytes at stages V-VI were selected and placed in Modified Barth's Medium (MBS) containing (in mM): 88 NaCl, 1 KCl, 2.4 NaHCO_3_ 15 HEPES-NaOH (pH 7.6), 0.33 Ca(NO_3_)_2_ · 4H_2_0, 0.41 CaCl_2_ · 6H2O, 0.82 MgSO_4_ · 7H_2_O, 2.5 Pyruvate, 10 µg/ml penicillin and 10 µg/ml streptomycin and were stored at +8°C.

### Microscopy

The oocytes were photographed using a USB-microscope (Dino-Lite Long AM4013TL, AnMo Electronics Corp., Taiwan)

### Electrophysiological recordings

Oocytes were incubated in MBS solution with (apoptosis) or without (control) 1 µl STS (Sigma-Aldrich, St Louis, MO) for 6 7 hours in room temperature before the electrophysiological measurements. The NaCl in the MBS solution were replaced either by 88 mM choline chloride (ChCl), 88 mM Tetramethylammonium chloride (TMA) or 88 mM KCl in certain experiments.

Ion currents were recorded by the two-electrode voltage-clamp technique (CA-1B amplifier, Dagan Corporation, Minneapolis, MN). The currents were low pass filtered at 5 kHz. The recordings were done in 100Na solution (in mM: 88 NaCl, 1 KCl, 15 HEPES, 0.4 CaCl_2_ and 0.8 MgCl_2_, pH adjusted to 7.4 with NaOH) or in a high K^+^, 0Na, solution (in mM: 89 KCl, 15 HEPES, 0.4 CaCl_2_ and 0.8 MgCl_2_, pH adjusted to 7.4 with KOH). The holding potential was set to −80 mV and the experiments were performed in 21 voltage steps of 10 mV to +100 mV. Tail currents were recorded at 0 mV after a 1 s prepulse to +100 mV. 100Na solutions containing TTX (1 µM), amiloride (10 µM) or verapamil (1, 10, 100 and 200 µM) were applied to the oocytes. In some experiments, NaCl was replaced by ChCl in the 100Na solution and the pH was set to 7.4 with TEAOH.

### Caspase-3 activity

Oocytes were incubated in MBS solution either 1 with 1 µM STS (apoptosis) for different durations (between one and 24 hours) or without STS (control). The NaCl in the MBS solution was replaced either by 88 mM ChCl, 88 mM TMA or 88 mM KCl in certain experiments. The oocytes were then washed in ELB buffer (in mM: 250 sucrose, 50 KCl, 10 HEPES (pH 7.7) and 2.5 MgCl_2_). The oocytes were lysed with lysis buffer (in mM: 130 NaCl, 10 NaH_2_PO_4_/NaHPO_4_, 10 Tris-HCl (pH 7.5), 10 sodium pyrophosphate and 1% Triton) and then centrifuged at 12000x g for 15 min. Reaction buffer (in mM: 20 HEPES (pH 7.5), 2 dithiothreitol, 0.02 Ac-DEVD-AMC (Becton, Dickinson and Company, Franklin Lakes, NJ) and 10% glycerol) and cytosolic fraction of the oocytes was added in 96-well plate and incubated in 37°C for 1 h. Fluorescence was measured using Perkin Elmer multilabel counter VICTOR^3^ V using the Wallace 1420 software version 3.00 (Perkin Elmer, Sweden) at the wavelengths 380/460 nm.

### Data analysis

All recordings were off-line leakage compensated for by the Clampfit software 10.4 (Axon Instruments/Molecular Devices, CA). The conductance, G, was calculated as
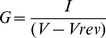
(1)where I is the ion current, V the absolute membrane voltage, and Vrev the reversal potential for the channel to be studied (measured directly from the experiments). Conductance versus voltage, G(V), was fitted to
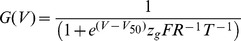
(2)where V50 is the voltage at which 50% of the channels reside in the open state, and where zg is the gating charge, that is the amount of charge that have to be moved to open the channel. R, T and F have their normal thermodynamic meanings. The intracellular Na+ concentration, [Na+]i, was calculated using Nernst's equation
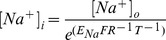
(3)where [Na+]o is the extracellular Na+ concentration (100 mM) and ENa is the equilibrium potential for Na+. F, R, and T have their normal thermodynamic meanings

Data are presented as mean±S.E.M, with *n* as the number of oocytes investigated.

Statistical analysis was performed using one-way ANOVA with Bonferroni *post hoc* tests and unpaired t-test (GraphPad Prism, software 5). Statistical significance was defined as p<0.05 (*), 0.01 (**) and 0.0001 (****).

## References

[1] Ulukaya E, Acilan C, Yilmaz Y (2011) Apoptosis: why and how does it occur in biology? Cell Biochem Funct 29: 468–480 10.1002/cbf.1774 21773978

[2] Arrebola F, Zabiti S, Cañizares FJ, Cubero MA, Crespo PV, et al (2005) Changes in intracellular sodium, chlorine, and potassium concentrations in staurosporine-induced apoptosis. J Cell Physiol 204: 500–507 10.1002/jcp.20306 15717314

[3] Arrebola F, Cañizares J, Cubero MA, Crespo PV, Warley A, et al (2005) Biphasic behavior of changes in elemental composition during staurosporine-induced apoptosis. Apoptosis 10: 1317–1331 10.1007/s10495-005-2718-x 16215671

[4] Yu SP, Yeh C-H, Sensi SL, Gwag BJ, Canzoniero LMT, et al (1997) Mediation of Neuronal Apoptosis by Enhancement of Outward Potassium Current. Science 278: 114–117 10.1126/science.278.5335.114 9311914

[5] Hughes FM, Bortner CD, Purdy GD, Cidlowski JA (1997) Intracellular K+ suppresses the activation of apoptosis in lymphocytes. J Biol Chem 272: 30567–30576. 9374553 10.1074/jbc.272.48.30567

[6] Singleton KR, Will DS, Schotanus MP, Haarsma LD, Koetje LR, et al (2009) Elevated extracellular K+ inhibits apoptosis of corneal epithelial cells exposed to UV-B radiation. Exp Eye Res 89: 140–151 10.1016/j.exer.2009.02.023 19289117

[7] Bortner CD, Cidlowski JA (2002) Apoptotic volume decrease and the incredible shrinking cell. Cell Death Differ 9: 1307–1310 10.1038/sj.cdd.4401126 12478467

[8] Wei L, Xiao AY, Jin C, Yang A, Lu ZY, et al (2004) Effects of chloride and potassium channel blockers on apoptotic cell shrinkage and apoptosis in cortical neurons. Pflugers Arch 448: 325–334 10.1007/s00424-004-1277-2 15057559

[9] Krumschnabel G, Maehr T, Nawaz M, Schwarzbaum PJ, Manzl C (2007) Staurosporine-induced cell death in salmonid cells: the role of apoptotic volume decrease, ion fluxes and MAP kinase signaling. Apoptosis 12: 1755–1768 10.1007/s10495-007-0103-7 17624593

[10] Akanda N, Tofighi R, Brask J, Tamm C, Elinder F, et al (2008) Voltage-dependent anion channels (VDAC) in the plasma membrane play a critical role in apoptosis in differentiated hippocampal neurons but not in neural stem cells. Cell Cycle 7: 3225–3234. 18927501 10.4161/cc.7.20.6831

[11] Bortner CD, Gómez-Angelats M, Cidlowski JA (2001) Plasma Membrane Depolarization without Repolarization Is an Early Molecular Event in Anti-Fas-induced Apoptosis. J Biol Chem 276: 4304–4314 10.1074/jbc.M005171200 11050080

[12] Bortner CD, Cidlowski JA (2003) Uncoupling cell shrinkage from apoptosis reveals that Na+ influx is required for volume loss during programmed cell death. J Biol Chem 278: 39176–39184 10.1074/jbc.M303516200 12821680

[13] Thompson GJ, Langlais C, Cain K, Conley EC, Cohen GM (2001) Elevated extracellular [K+] inhibits death-receptor- and chemical-mediated apoptosis prior to caspase activation and cytochrome c release. Biochem J 357: 137–145. 11415444 10.1042/0264-6021:3570137 PMC1221936

[14] Banasiak KJ, Burenkova O, Haddad GG (2004) Activation of voltage-sensitive sodium channels during oxygen deprivation leads to apoptotic neuronal death. Neuroscience 126: 31–44 10.1016/S0306-4522(03)00425-1 15145071

[15] Elinder F, Akanda N, Tofighi R, Shimizu S, Tsujimoto Y, et al (2005) Opening of plasma membrane voltage-dependent anion channels (VDAC) precedes caspase activation in neuronal apoptosis induced by toxic stimuli. Cell Death Differ 12: 1134–1140 10.1038/sj.cdd.4401646 15861186

[16] Akanda N, Elinder F (2006) Biophysical properties of the apoptosis-inducing plasma membrane voltage-dependent anion channel. Biophys J 90: 4405–4417 10.1529/biophysj.105.080028 16581845 PMC1471872

[17] Armstrong CM (2003) The Na/K pump, Cl ion, and osmotic stabilization of cells. Proc Natl Acad Sci USA 100: 6257–6262 10.1073/pnas.0931278100 12730376 PMC156359

[18] Maeno E, Ishizaki Y, Kanaseki T, Hazama A, Okada Y (2000) Normotonic cell shrinkage because of disordered volume regulation is an early prerequisite to apoptosis. Proc Natl Acad Sci USA 97: 9487–9492 10.1073/pnas.140216197 10900263 PMC16891

[19] Johnson CE, Freel CD, Kornbluth S (2010) Features of programmed cell death in intact Xenopus oocytes and early embryos revealed by near-infrared fluorescence and real-time monitoring. Cell Death Differ 17: 170–179 10.1038/cdd.2009.120 19730443 PMC2794955

[20] Nutt LK, Margolis SS, Jensen M, Herman CE, Dunphy WG, et al (2005) Metabolic regulation of oocyte cell death through the CaMKII-mediated phosphorylation of caspase-2. Cell 123: 89–103 10.1016/j.cell.2005.07.032 16213215 PMC2788768

[21] Ali S, Asif M, Fulati A, Nur O, Willander M, et al.. (2010) Intracellular K+ Determination With a Potentiometric Microelectrode Based on ZnO Nanowires. Nanotechnology, IEEE Transactions on PP: 1. doi:10.1109/TNANO.2010.2089696.

[22] Börjesson SI, Englund UH, Asif MH, Willander M, Elinder F (2011) Intracellular K+ concentration decrease is not obligatory for apoptosis. J Biol Chem 286: 39823–39828 10.1074/jbc.M111.262725 21949184 PMC3220575

[23] Abelson JN, Simon MI, Rudy B, Iverson LE, editors (1997) Ion Channels, Volume 207 (Methods in Enzymology), Vol 207. Academic Press. 917 p.

[24] Asif MH, Fulati A, Nur O, Willander M, Brannmark C, et al.. (2009) Functionalized zinc oxide nanorod with ionophore-membrane coating as an intracellular Ca2+ selective sensor. Applied Physics Letters 95: 023703 –023703–3. doi:10.1063/1.3176441.

[25] Hille B (2001) Ion Channels of Excitable Membranes. 3rd Edition. Sinauer Associates.

[26] Dib-Hajj S, Black JA, Cummins TR, Waxman SG (2002) NaN/Nav1.9: a sodium channel with unique properties. Trends in Neurosciences 25: 253–259 10.1016/S0166-2236(02)02150-1 11972962

[27] Rolf S, Haverkamp W, Borggrefe M, Musshoff U, Eckardt L, et al (2000) Effects of antiarrhythmic drugs on cloned cardiac voltage-gated potassium channels expressed in Xenopus oocytes. Naunyn Schmiedebergs Arch Pharmacol 362: 22–31. 10935529 10.1007/s002100000257

[28] Madeja M, Müller V, Musshoff U, Speckmann EJ (2000) Sensitivity of native and cloned hippocampal delayed-rectifier potassium channels to verapamil. Neuropharmacology 39: 202–210. 10670415 10.1016/s0028-3908(99)00110-0

[29] Yamagishi T, Ishii K, Taira N (1995) Antiarrhythmic and bradycardic drugs inhibit currents of cloned K+ channels, KV1.2 and KV1.4. Eur J Pharmacol 281: 151–159. 7589202 10.1016/0014-2999(95)00240-l

[30] Roger S, Le Guennec J-Y, Besson P (2004) Particular sensitivity to calcium channel blockers of the fast inward voltage-dependent sodium current involved in the invasive properties of a metastastic breast cancer cell line. Br J Pharmacol 141: 610–615 10.1038/sj.bjp.0705649 14744811 PMC1574233

[31] Hodgkin AL, Huxley AF (1952) Currents carried by sodium and potassium ions through the membrane of the giant axon of Loligo. J Physiol (Lond) 116: 449–472. 14946713 10.1113/jphysiol.1952.sp004717 PMC1392213

[32] Baud C, Kado RT, Marcher K (1982) Sodium channels induced by depolarization of the Xenopus laevis oocyte. Proc Natl Acad Sci USA 79: 3188–3192. 6285341 10.1073/pnas.79.10.3188 PMC346380

[33] Dascal N (1987) The Use of Xenopus Oocytes for the Study of Ion Channel. Crit Rev Biochem Mol Biol 22: 317–387 10.3109/10409238709086960 2449311

[34] Asif MH, Nur O, Willander M, Strålfors P, Brännmark C, et al (2010) Growth and Structure of ZnO Nanorods on a Sub-Micrometer Glass Pipette and Their Application as Intracellular Potentiometric Selective Ion Sensors. Materials 3: 4657–4667 10.3390/ma3094657 28883346 PMC5445763

[35] Weber WM, Liebold KM, Clauss W (1995) Amiloride-sensitive Na+ conductance in native Xenopus oocytes. Biochim Biophys Acta 1239: 201–206. 7488625 10.1016/0005-2736(95)00151-r

[36] Kupitz Y, Atlas D (1993) A putative ATP-activated Na+ channel involved in sperm-induced fertilization. Science 261: 484–486. 8392753 10.1126/science.8392753

[37] Burckhardt BC, Burckhardt G (1997) NH4+ conductance in Xenopus laevis oocytes. I. Basic observations. Pflugers Arch 434: 306–312. 9178631 10.1007/s004240050401

[38] Vasilyev A, Indyk E, Rakowski RF (2002) Properties of a Sodium Channel (Nax) Activated by Strong Depolarization of Xenopus Oocytes. J Membr Biol 185: 237–247 10.1007/s00232-001-0126-x 11891581

[39] Charpentier G (2002) Effect of lidocaine on the slow Na+ channels of Xenopus oocytes. Gen Physiol Biophys 21: 355–365. 12693709

[40] Yang D, Yang D, Jia R, Ding G (2013) Selective inhibition of the reverse mode of Na(+)/Ca(2+) exchanger attenuates contrast-induced cell injury. Am J Nephrol 37: 264–273 10.1159/000348526 23485664

[41] Liu W, Zhao H, Wang Y, Jiang C, Xia P, et al (2013) Calcium-calmodulin signaling elicits mitochondrial dysfunction and the release of cytochrome c during cadmium-induced apoptosis in primary osteoblasts. Toxicol Lett 224: 1–6 10.1016/j.toxlet.2013.10.009 24144892

[42] Khananshvili D (2013) Sodium-calcium exchangers (NCX): molecular hallmarks underlying the tissue-specific and systemic functions. Pflugers Arch. doi:10.1007/s00424-013-1405-y. 10.1007/s00424-013-1405-y 24281864

[43] Solís-Garrido LM, Pintado AJ, Andrés-Mateos E, Figueroa M, Matute C, et al (2004) Cross-talk between native plasmalemmal Na+/Ca2+ exchanger and inositol 1,4,5-trisphosphate-sensitive ca2+ internal store in Xenopus oocytes. J Biol Chem 279: 52414–52424 10.1074/jbc.M408872200 15375168

[44] Hirn C, Shapovalov G, Petermann O, Roulet E, Ruegg UT (2008) Nav1.4 deregulation in dystrophic skeletal muscle leads to Na+ overload and enhanced cell death. J Gen Physiol 132: 199–208 10.1085/jgp.200810024 18625851 PMC2483333

[45] Poulsen KA, Andersen EC, Hansen CF, Klausen TK, Hougaard C, et al (2010) Deregulation of apoptotic volume decrease and ionic movements in multidrug-resistant tumor cells: role of chloride channels. Am J Physiol, Cell Physiol 298: C14–25 10.1152/ajpcell.00654.2008 19846756

